# Transcatheter closure of a congenital coronary artery to right ventricle fistula: a case report

**DOI:** 10.1186/1752-1947-8-432

**Published:** 2014-12-16

**Authors:** Rym Gribaa, Mehdi Slim, Sana Ouali, Elies Neffati, Essia Boughzela

**Affiliations:** Department of Cardiology, Sahloul University Hospital, Hammam Sousse, Route de la Ceinture, 4011 Sousse, Tunisia

**Keywords:** Congenital heart disease, Coronary artery fistula, Transcatheter therapy

## Abstract

**Introduction:**

Congenital coronary artery fistula is a rare anomaly that may cause angina, atrial fibrillation, endocarditis, aneurysmal dilation and myocardial infarction. Both spontaneous regression and life-threatening complications have been described. Treatment can be conservative, surgical or more recently through transcatheter closure.

**Case presentation:**

We report the case of a 27-year-old Tunisian man with a large coronary artery fistula from the left anterior descending artery to the right ventricle associated with pulmonary stenosis. This patient underwent a successful transcatheter closure of his coronary artery fistula followed by pulmonary dilatation and had an uneventful recovery after treatment.

**Conclusions:**

Transcatheter closure of a congenital coronary artery fistula is feasible and should be considered in carefully selected patients. Recanalization of the treated coronary fistula can occur, so follow-up angiography or other imaging modality should be performed in these patients.

## Introduction

Congenital coronary artery fistulas (CAFs) are abnormal connections between either or both coronary arteries and a cardiac chamber or a great vessel. The drainage of these fistulas is more typically to the right atrium, right ventricle, or right atrium-superior vena cava junction, and occasionally to the coronary sinus or left side of the heart. A congenital CAF is a rare anomaly with a 0.2% to 0.6% incidence in angiographic series and 0.002% overall incidence in the general population [1, 2]. Congenital CAFs can be single or multiple, and may be associated with other cardiac abnormalities. Approximately 10% to 30% of patients with a CAF also have another congenital cardiovascular anomaly [3, 4]. The most commonly seen defects include variations of tetralogy of Fallot, patent ductus arteriosus, atrial septal defect, ventricular septal defect and pulmonary stenosis. Conventional coronary angiography is a commonly used diagnostic modality for tracing the anatomic course of coronary fistulas. Computed tomography (CT) angiography is also an important tool for defining size, anatomy and the relation of the CAFs to adjacent structures [5]. Most patients are asymptomatic. However, a CAF may cause heart failure secondary to volume overload resulting from left to right shunting, ischemia secondary to coronary steal, arrhythmia, fistula rupture or thrombosis, and infective endocarditis [6]. That is why closure of large CAFs is indicated to prevent these complications. The American College of Cardiology/American Heart Association (2008) guidelines for the management of adults with congenital heart disease recommend closure of all large CAFs, regardless of symptoms, using transcatheter or surgical techniques [7]. Since the first percutaneous closure performed in 1983 [8], numerous reports of transcatheter closure have been described. We report the case of a patient who underwent successful transcatheter closure of a CAF followed by pulmonary dilatation.

## Case presentation

**Figure 1:**
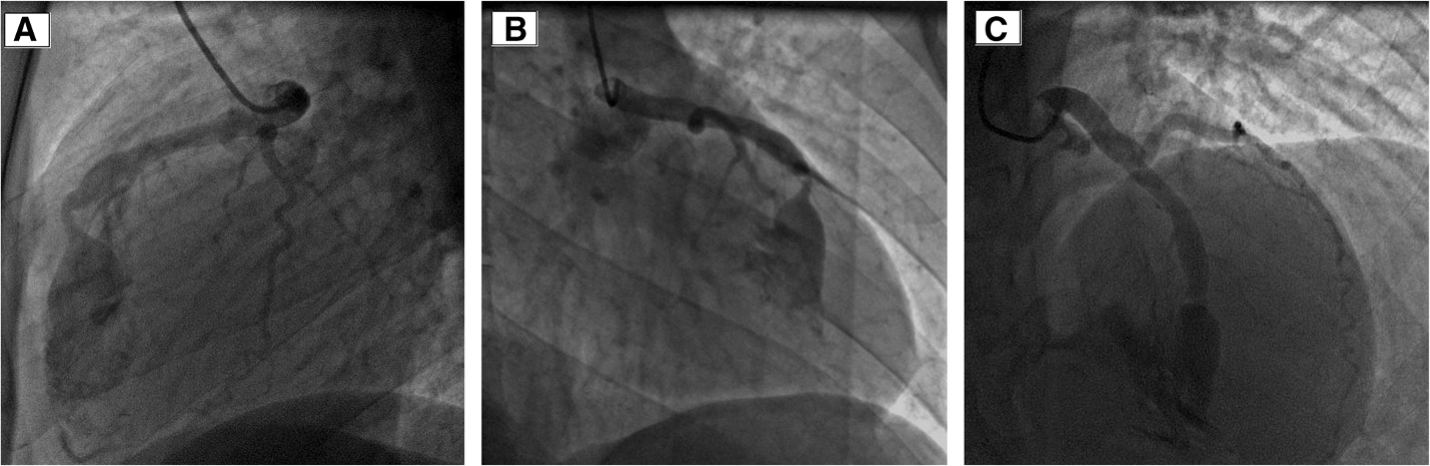
**Selective coronary angiography showing a coronary artery fistula arising from the segment of the left anterior descending artery draining into the right ventricle.** Anterior oblique 90 degree view **(Panel 1A)**. Right anterior oblique 38 degree view **(Panel 1B)**. Cranial 38 degree and right anterior oblique 12 degree view **(Panel 1C)**.

**Figure 2:**
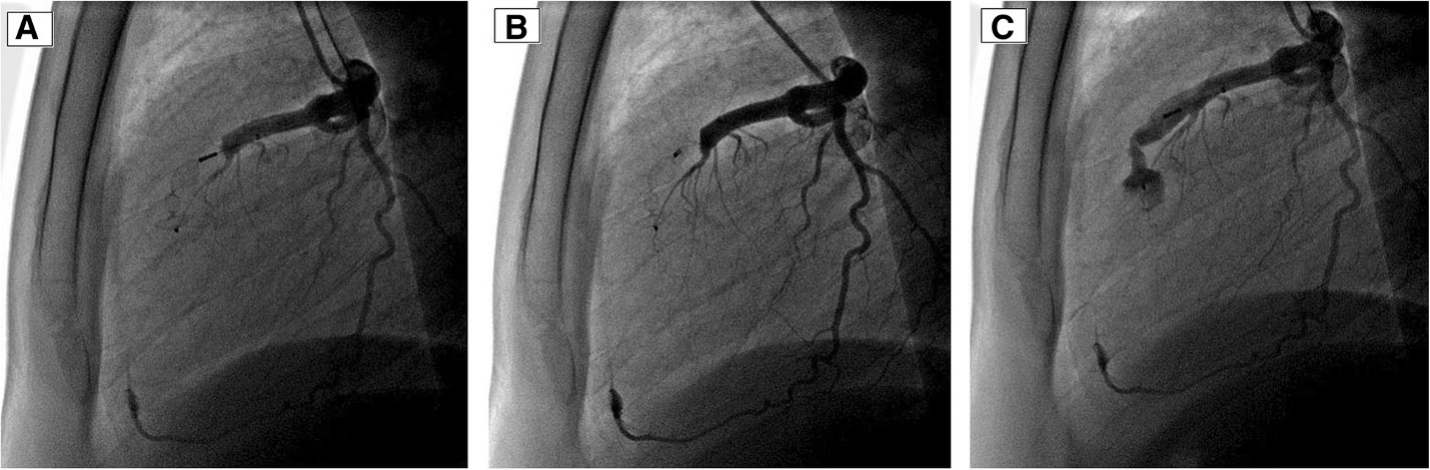
**Transcatheter closure of the fistula.** The Amplatzer™ duct occluder is shown in place while it is still connected to the delivery guidewire (left anterior oblique view) **(Panel 2A)**. Angiography confirming complete occlusion following deployment of the Amplatzer™ Duct Occluder (left anterior oblique view) **(Panel 2B)**. Angiography showing the device in place and residual flow through the device (left anterior oblique view) **(Panel 2C)**.

We report the case of a 27-year-old Tunisian man who presented with dyspnea (New York Heart Association (NYHA) functional class II) associated with typical angina. He has a medical history of ventricular septal defect associated with pulmonary stenosis diagnosed at 15 years old, with surgical treatment refused by his parents and with interrupted follow-up since then. Currently, a physical examination was notable for a grade 3/6 systolic murmur over the second left intercostal space without signs of heart failure or cyanosis. His electrocardiogram showed sinus rhythm with incomplete right bundle branch block. His chest X-ray showed cardiomegaly and dilatation of his left pulmonary artery. Transthoracic echocardiography showed normal left ventricular systolic function without wall motion abnormalities. The right ventricle was hypertrophied, dilated but with normal contractility. Severe pulmonary valvular stenosis was also visualized. An exploration of the ventricular septum showed a closed ventricular septal defect without evidence of shunt, related to a spontaneous closure of the defect. Cardiac catheterization confirmed a tight valvular pulmonary stenosis with systolic right ventricular and pulmonary arterial pressures at 103 and 14mmHg respectively, resulting in a 95mmHg systolic gradient between the right ventricle and pulmonary artery. Selective pulmonary angiography showed a severe valvular pulmonary stenosis and a left pulmonary artery stenosis with a poststenotic aneurysm. Left ventricle angiography showed a 12mm ventricular septal defect completely closed and an aneurismal left coronary artery. Selective coronary angiography revealed a dilated left coronary artery and 3mm coronary fistula arising from the mid segment of the left anterior descending artery (LAD) to the right ventricle (RV) with occlusion of the distal LAD (Figure 1). Percutaneous treatment of both lesions was decided starting with the CAF to prevent acute myocardial ischemia that may appear after acute coronary steal consequent to the decrease of the RV pressure after relief of the pulmonary obstruction. A successful transcatheter closure of the fistula using a 4/6 Amplatzer™ duct occluder II (ADO II) was achieved, with partial reopening of the fistula on the following day (Figure 2). No electrocardiogram changes were observed during or after the procedure. Heparin therapy was started with 150mg aspirin and 75mg clopidogrel to prevent thrombosis of the occluded vessel and discontinued after evidence of partial reopening of the fistula on systematic echocardiography control performed 24 hours after the procedure.

**Figure 3:**
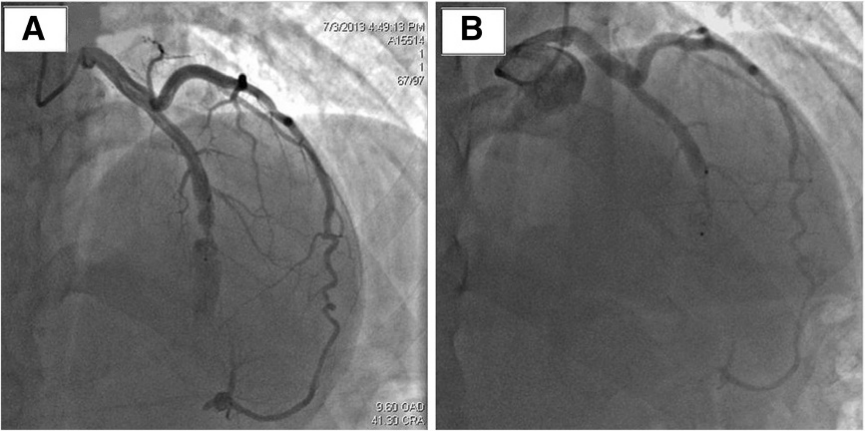
**Angiography showing residual flow through the device.** Cranial 38 degree and right anterior oblique 12 degree view **(Panel 3A).** The same angiographic view showing complete occlusion of the fistula after nine months **(Panel 3B)**.

Serial electrocardiograms documented normal tracings without evidence of ischemia. Echocardiography showed normal left ventricular function with no pericardial effusion, allowing discharge 48 hours after the procedure. Our patient was on 150mg aspirin per day. Complete closure of the fistula was documented nine months later (Figure 3) allowing a successful percutaneous treatment of the pulmonary stenosis, with reduction of the pulmonary gradient from 100 to 35mmHg, using a 23/40mm balloon, with an uneventful recovery after treatment.

## Discussion

The management strategy of patients with a CAF depends on the size and the anatomy of the fistula, presence of symptoms, the patient’s age and whether the patient has other associated cardiovascular disorders. As recommended by the American College of Cardiology/American Heart Association (2008) guidelines [7], small fistulas discovered incidentally in asymptomatic patients should not be treated. However, periodic clinical evaluation with imaging such as echocardiography to assess both the size of the fistula and ventricular function is indicated. In fact, small fistulas may slowly increase in size with advancing age and changes in systemic blood pressure and aortic compliance and may subsequently require treatment [9]. Large and small-to-moderate congenital CAFs with suitable anatomy, without other complex cardiac lesions and in the presence of symptoms of myocardial ischemia, threatening arrhythmia, unexplained ventricular dysfunction, or left atrial hypertension, must be closed using transcatheter or surgical techniques. The first successful surgical closure was reported by Biork *et al.* [10] in 1947.

Since then, surgical closure of a CAF is the only option but should be performed by surgeons with training and expertise [7]. The disadvantages of surgery are bleeding, postpericardiotomy syndrome, myocardial infarction and recurrence. Furthermore, surgery requires a median sternotomy and sometimes cardiopulmonary bypass. In the surgical literature [3], the rate of mortality is low (1%), cardiopulmonary bypass was used in approximately one-half of the cases [11, 12], the rate of incomplete closure or recurrence of fistulas was approximately 10%. Cheung *et al*. reported a series of 41 patients [3] who had CAFs treated by surgery, 21 patients had undergone cardiac catheterization in the follow-up, four (19%) of them had residual or recurrent fistulas. Other rare complications, including arrhythmias, myocardial infarctions, stroke, and ST-/T-wave changes on the electrocardiogram, were reported. The first transcatheter closure was performed by Reidy *et al.* [8] in 1983. Since then numerous reports of transcatheter closure have been described [13, 14]. Criteria for transcatheter closure are similar to those used for surgical closure. It should be done only in centers with particular expertise in such interventions [7]. The approach involves several techniques and devices, including coils, detachable balloons, covered stents, vascular plugs, and atrial septal defect devices [15], but controversy remains as to the best approach. In comparison with many devices used to close CAFs, the Amplatzer™ duct occluder (ADO) has several advantages, including the use of a single device, a high rate of complete occlusion, and improved control over placement and release of the device. The ADO II is a modification of the ADO I and has several advantages over it. The device stretches to accommodate different lengths and angles. It is a flexible and low-profile occluder (composed of multiple layers of fine nitinol braiding without fabric), highly conformable to the anatomy (with articulating ‘necks’), offers secure device placement, and uses low-profile delivery catheters (4 and 5 Fr) with high trackability [16]. For these reasons, we opted for the ADO II device.

The advantages of transcatheter closure include less morbidity, lower cost, shorter recovery time, and avoidance of thoracotomy and cardiopulmonary bypass [13].

The procedural complications include transient ischemic changes, unretrieved device embolization, fistula dissection, myocardial infarction, and transient atrial arrhythmia [14]. In our case, the choice between transcatheter closure of the fistula and surgical intervention was controversial. Despite the association of fistula and severe pulmonary stenosis, we treated our patient using the transcatheter technique. In fact, the anatomy of the fistula and the valvular pulmonary stenosis were suitable for percutaneous treatment, a more low-risk procedure than surgery.

The transcatheter pulmonary dilatation was performed after complete regression of the residual shunt. In the literature, several reports have evaluated the efficacy and safety of transcatheter closure of a CAF. Although, most of the series report an excellent procedural success rate, there is limited information about the short- and long-term prognosis of transcatheter-treated patients with a CAF [15, 17]. Armsby and colleagues reported short-term findings in 33 patients who underwent transcatheter closure [16]. After median follow-up, only five patients had small residual shunts. Zhu *et al.* [18] reported transcatheter closure results of 24 patients. All patients were asymptomatic with complete closure of the CAF except for one patient (5%), who had a recurrence of shunt at six-month follow-up, which was re-closed by percutaneous technique. Jama *et al.* [15] reported transcatheter closure results of 29 patients with immediate successful closure in all patients. Ten (56%) of the 18 patients with follow-up angiography had no recanalization of embolized vessels; four patients (22%) had trivial recanalization, and four patients (22%) had large recanalizations, which were re-closed by percutaneous technique. In our case, we performed an immediate successful closure of the fistula without any procedural complication. The early recurrence of mild shunt was due to the combination of heparin and double platelet aggregation inhibitors during the first 24 hours of the procedure. This initial residual shunting disappeared completely during the follow-up.

## Conclusions

The successful results of the percutaneous closure of coronary artery fistula in our patient suggest that this technique is feasible and is a safe alternative to surgical ligation. The standardization of this approach requires study of a greater number of cases with longer follow-up periods.

## Consent

Written informed consent was obtained from the patient for publication of this case report and any accompanying images. A copy of the written consent is available for review by the Editor-in-Chief of this journal.

## Abbreviations

ADO: Amplatzer™ duct occluder
CAF: coronary artery fistula
CT: computed tomography
LAD: left anterior descending artery
RV: right ventricle.
